# Interferons and Resistance Mechanisms in Tumors and Pathogen-Driven Diseases—Focus on the Major Histocompatibility Complex (MHC) Antigen Processing Pathway

**DOI:** 10.3390/ijms24076736

**Published:** 2023-04-04

**Authors:** Chiara Massa, Yuan Wang, Nico Marr, Barbara Seliger

**Affiliations:** 1 Medical Faculty, Martin Luther University Halle-Wittenberg, Magdeburger Str. 2, 06112 Halle, Germany; 2 Institute for Translational Immunology, Brandenburg Medical School Theodor Fontane, Hochstr. 29, 14770 Brandenburg an der Havel, Germany; 3 College of Health and Life Sciences, Hamad Bin Khalifa University, Doha P.O. Box 34110, Qatar; 4 Fraunhofer Institute for Cell Therapy and Immunology, Perlickstr. 1, 04103 Leipzig, Germany

**Keywords:** interferon, tumor, MHC class I, therapy

## Abstract

Interferons (IFNs), divided into type I, type II, and type III IFNs represent proteins that are secreted from cells in response to various stimuli and provide important information for understanding the evolution, structure, and function of the immune system, as well as the signaling pathways of other cytokines and their receptors. They exert comparable, but also distinct physiologic and pathophysiologic activities accompanied by pleiotropic effects, such as the modulation of host responses against bacterial and viral infections, tumor surveillance, innate and adaptive immune responses. IFNs were the first cytokines used for the treatment of tumor patients including hairy leukemia, renal cell carcinoma, and melanoma. However, tumor cells often develop a transient or permanent resistance to IFNs, which has been linked to the escape of tumor cells and unresponsiveness to immunotherapies. In addition, loss-of-function mutations in IFN signaling components have been associated with susceptibility to infectious diseases, such as COVID-19 and mycobacterial infections. In this review, we summarize general features of the three IFN families and their function, the expression and activity of the different IFN signal transduction pathways, and their role in tumor immune evasion and pathogen clearance, with links to alterations in the major histocompatibility complex (MHC) class I and II antigen processing machinery (APM). In addition, we discuss insights regarding the clinical applications of IFNs alone or in combination with other therapeutic options including immunotherapies as well as strategies reversing the deficient IFN signaling. Therefore, this review provides an overview on the function and clinical relevance of the different IFN family members, with a specific focus on the MHC pathways in cancers and infections and their contribution to immune escape of tumors.

## 1. Important Features of the Family of Interferons

Interferons (IFNs) are a family of multi-functional cytokines, which were originally described as anti-viral mediators protecting cells from viral infection [1]. However, based on the current knowledge, IFNs exhibit a broad spectrum of activities including anti-proliferative, immunomodulatory, anti-inflammatory, apoptosis-inducing, stress-mediated effects, as well as the regulation of cell differentiation and angiogenesis [2,3,4]. Based on the degree of sequence homology, the IFN family has been divided into type I, type II, and type III IFNs [5] (Table 1). In humans, type I IFNs comprise 13 IFN-α subtypes, IFN-β, and several less characterized gene products, including IFN-ĸ, IFN-Ω, and IFN-ε, which are all clustered in humans on chromosome 9 [6]. Despite a redundancy, differences in promoter sequences and biochemical properties contribute to the partially distinct functional activities of type I IFNs [7]. In contrast, type II IFN is only represented by one single protein, IFN-γ, encoded in humans on chromosome 12 [8]. Recently, type III IFNs, also known as interleukin (IL)-28A, IL-28B, and IL-29, were discovered as a novel class of anti-viral cytokines and classified into IFN-λ1, -λ2, and -λ3 [9,10]. Moreover, some humans encode a functional IFN-λ4 protein, whereas in others, *IFNL4* is a pseudogene [11]. The genes encoding this type of IFNs are clustered together on human chromosome 19 [9,12,13,14]. In general, IFNs bind to distinct, species-specific cell surface receptors (IFNRs) [5,15]. All type I IFNs share a heterodimeric receptor (IFNAR) consisting of the IFNAR1 and IFNAR2 subunits with a common γ chain leading to the activation of distinct, but also related pathways. All type III IFN subtypes bind to the heterodimeric IFNLR consisting of the IL-10RB and IFN-λR chain (IL-28RA), the unique and specific receptor chain for all IFN-λs, suggesting that IFN-λ is related to both IFN by sharing similar signaling and activities of type I IFNs and proteins of the IL-10 family. IFN-γ binds to its own receptor consisting of the IFNGR1 and IFNGR2 subunits [16].

## 2. Interferon Signal Transduction Pathways and Their Components

**Figure 1:**
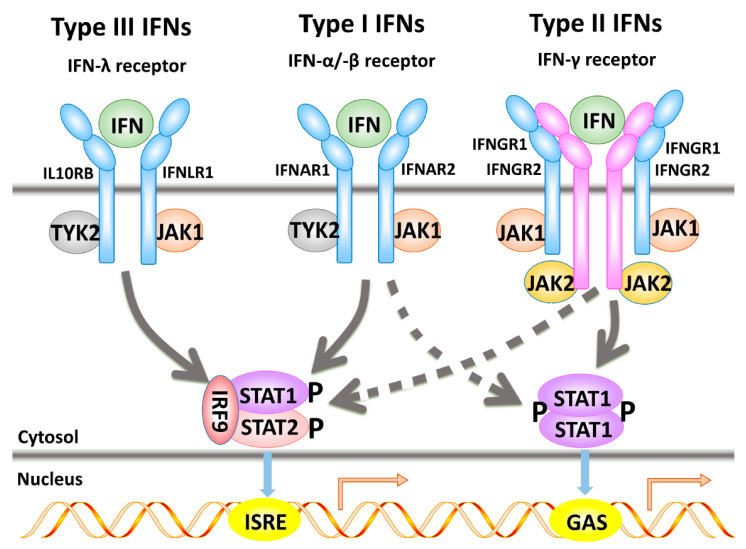
Interferon signaling pathways. Dotted arrows indicate crosstalk between the downstream signaling pathways.

IFN signaling represents the basis of innate immune responses against diverse pathogens and tumors [17]. During the last decades, a wealth of information has become available on the molecular processes underlying the IFN-induced signaling cascades and their contribution to the function of IFNs. Engagement of IFNs to their specific receptors, which lack intrinsic kinase activities, induces a crosslink between the receptor subunits followed by the activation of distinct, but related pathways [18] (Figure 1). This results in the transcriptional activation of a plethora of target genes, the so-called IFN-stimulated genes (ISGs), which represent a functionally diverse group of hundreds of genes involved in transcription, translation, regulation of cell cycle and apoptosis, intracellular communication, as well as the processing and presentation of antigens (Ag) [19,20,21,22]. Despite the existence of different IFNRs, the spatiotemporal dynamics of IFN responses and ligand-receptor binding kinetics, the downstream signaling events and transcriptional regulation exhibit a substantial overlap [23] (Figure 1).

The physiological relevance of the IFN-dependent signal transduction cascades, in particular, the signal transducer and activator of transcription (STAT) and janus kinase (JAK) pathway, was determined by the generation and characterization of mice with targeted disruptions of genes encoding, e.g., IFNRs, STAT1/STAT2 or JAK1, respectively [24,25]. The IFN/IFNR interaction initiates the activation of the JAK/STAT cascade, which consists of the four janus kinases JAK1, JAK2, JAK3, and JAK4 and seven signal transducers and activators of transcription, namely STAT1, STAT2, STAT3, STAT4, STAT5a, STAT5b, and STAT5c [26,27]. The STATs represent cytosolic proteins of 750 to 800 amino acids with (i) an N-terminal domain, (ii) a ligand binding domain, and (iii) a C-terminal transactivation domain and exert different functions [23]. For example, STAT1 can suppress the growth and invasion of tumors, whereas STAT3 inhibits apoptosis and induces tumor cell proliferation and tumor-promoting inflammation [28]. However, the activation of the JAK-STAT pathway alone is not sufficient for the generation of the plethora of IFN activities. Indeed, several IFN-regulated signaling elements and cascades cooperate with STATs to optimize the transcriptional regulation of target genes, while others operate independently of the JAK-STAT pathway. These include signaling components linked to cellular stress and cell death, such as the mitogen-activated protein kinase [25,29], ERK, the stress-induced kinase p38 and protein kinase C, which are known to be involved in the different IFN-mediated signal transduction pathways [30,31,32], and due to their diversity, are responsible for the broad range of biological functions of IFNs. Despite the fact that different IFN signaling cascades have overlapping activities, the IFN family members also have significantly different features (Table 1) [33], which were recently confirmed by transcriptomic profiling of untreated vs. IFN-treated cells, resulting in the generation of diverse IFN signatures. 

### 2.1. Type I Interferon-α-Induced Signal Transduction Pathways

The binding of type I IFNs to the IFNAR triggers the activation of JAK1 and TYK2, leading to the phosphorylation of Tyr-466 of the IFN-αR1, which serves as a docking site for STAT2 [34]. The activated kinase subsequently phosphorylates STAT2 and STAT1 at residues Tyr-690 and Tyr-701, respectively. The two phosphorylated STATs form a heterodimer and associate with the IFN regulatory factor (IRF)9. This undergoes tyrosine phosphorylation to form the IFN-stimulated gene factor 3 (ISGF3), which in turn translocates to the nucleus and binds to the IFN-stimulated response elements (ISREs) present in the promoter region of certain ISGs, thereby initiating their transcription (reviewed in [35]). In addition, other STAT complexes, including STAT1 homodimers, and combinations of different STAT-containing complexes can be formed, which translocate to the nucleus and bind to the IFN-γ-activated sites (GAS), leading to the transcription of further genes [36]. IFN-α can also activate STAT3 and STAT5a/b, but the role of STAT5 in the IFN-α-mediated activity has still to be elucidated [37,38]. One may speculate that the fine balance between different STAT complexes might account for specific responses and represent a key mechanism for IFN-α-induced activities [39].

### 2.2. Type II Interferon-Induced Signal Transduction Cascade

Binding of IFN-γ initially leads to the formation of the IFNGR1 and IFNGR2 heterodimer, followed by signaling via the JAK/STAT pathway (reviewed in [40]). Upon activating JAK1 and JAK2, the JAKs phosphorylate the Tyr-440 at the intracellular domain of the IFNγR1, serving as a docking site for the latent cytosolic transcription factor STAT1. STAT1 is subsequently phosphorylated on Tyr-701 and Ser-727, leading to the homodimerization of phospho-STAT1 molecules [41]. These form a complex named the γ-activating factor that translocates into the nucleus and upregulates the transcription of IFN-γ-regulated genes, including IRF1 and IRF7 as transcriptional activators, whereas the constitutively expressed IRF2 generally acts as a transcriptional repressor [16,42]. In addition, IFN-γ may activate STAT5b [43,44].

### 2.3. Type III-Induced Interferon Signal Transduction

Similar to type I IFNs, type III IFNs signal through the cognate receptors that engage distinct JAKs and multiple downstream signaling cascades. This triggers the heterotrimeric transcription factor complex ISGF3 comprised of STAT1, STAT2, and IRF9 [5]. The activated ISGF3 translocates to the nucleus and binds to the ISREs in the upstream promoter regions of the ISGs [19,45,46]. However, in certain cell types, type III IFNs also activate the JAK2/AKT signaling, thereby altering the formation of reactive oxygen species [47,48].

### 2.4. Interferon Regulatory Factors

The interferon regulatory factors (IRFs) are effector transcription factors (TFs), in which distinct nucleic and sensing pathways converge. Alone or in complex with STATs, IRFs participate in different (patho) physiological processes [23]. Mammalian IRFs consist of nine family members (IRF1-9) [49] and contain an N-terminal conserved DNA-binding domain that can bind to the consensus ISRE. Despite their identification as transcriptional regulators of IFNs and IFN-inducible genes, IRFs also exhibit important functions in modulating immunity, oncogenesis, pathogen infections, and metabolism [23,50]. In particular, they are involved in the pathogenesis of associated diseases and mediate, e.g., constitutive and inducible host defenses against tumors and a variety of viruses and bacteria [23,51]. Their exact biological and immunological functions have been identified by the analysis of inborn errors of immunity as outlined in Section 4.5 and Section 4.6 [23].

## 3. The Major Histocompatibility Class I and Class II Antigen Processing and Presentation Pathways

### 3.1. General Features of the MHC Antigen Processing Machinery

**Figure 2:**
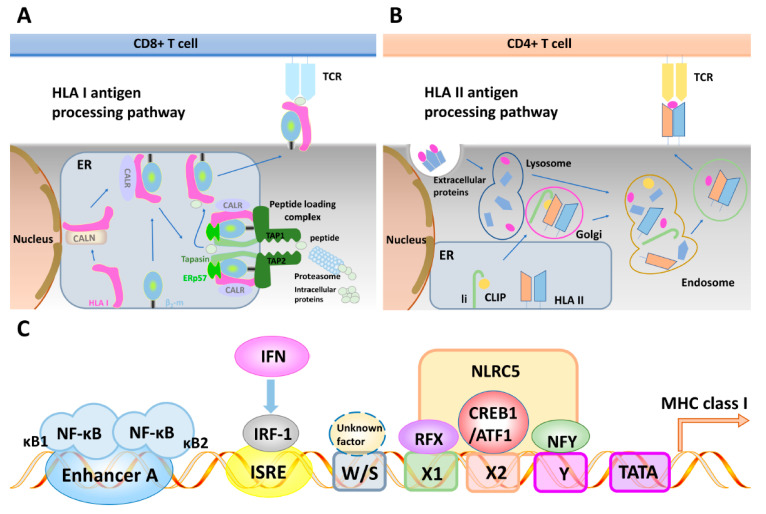
Schematic representation of the major histocompatibility complex class I and class II antigen processing components and their regulatory elements. (**A**) MHC class I heavy chain assembly with β_2_-m, which is assisted by various chaperones, such as calnexin and calreticulin. The MHC/β_2_-m dimer is incorporated into the peptide loading complex (PLC) in the ER. In the cytosol, endogenous peptides are generated by the proteasome, which are further trimmed by other peptidases, and then transported into the ER via the heterodimeric TAP. ERAP is involved in the final amino-terminal trimming of peptides. The loading of MHC class I molecules with peptides is further assisted by the chaperone tpn, which is also a component of the PLC. Upon peptide loading, the PLC dissociates, and is then transported via the trans Golgi to the cell surface, for engagement with the TCR of CD8^+^ cytotoxic T lymphocytes. (**B**) MHC class II molecules assemble in the ER with the invariant chain (li), which contains an endosomal targeting signal. This complex is then transported to the endosomal compartment where the li is cleaved by a number of proteases, leaving only the CLIP fragment, which occupies the peptide binding groove. HLA-DM and -DO catalyze the release of CLIP, which is exchanged by antigenic peptides. HLA-DM edits the repertoire of the MHC class II-peptide complexes, which are then transported to the cell surface for recognition by CD4^+^ T lymphocytes. Exogenous proteins are internalized into the endosomal pathway by different mechanisms, then unfolded and cleaved, which is catalyzed by different proteases. In addition, the yielded peptides are further trimmed after binding to MHC class II molecules. (**C**) Schematic illustration of the structure of representative promoters of the major APM components, depicting a number of transcription factor binding sides, such as NF-ĸB, AP1, SP1, and CREB, as well as interferon regulatory response elements (ISRE), which are involved in the transcriptional regulation.

The expression of the MHC class I and class II molecules is critical for the presentation of self and foreign antigens and essential for the generation of an adaptive T cell-mediated immune response [52]. During the last decades, CD8^+^ cytotoxic T lymphocytes (CTL) have been implicated as main effector cells in anti-tumor and anti-pathogen responses, since they were able to recognize and attack tumor or pathogen-infected cells presenting intracellular Ags derived from different non-self proteins on their surface through the interaction of the T cell receptor (TCR) with MHC class I peptide complexes [53]. The generation and presentation of these Ags require a coordinated expression of several genes (Figure 2A), as recently summarized [54]. Briefly, endogenously synthesized proteins are cleaved by the multicatalytic proteasome complex consisting of constitutive and IFN-γ regulated proteasome subunits, namely the low molecular weight proteins (LMP)2, LMP7, and LMP10 generating the correct N-terminus of the peptide, while these peptides can be further trimmed by cytosolic enzymes, such as, for example, the tripeptidyl peptidase II and the bleomycin hydrolase [55,56]. Then, the yielded peptides mainly of 8–10 amino acids in length are transported from the cytosol into the endoplasmic reticulum (ER) via the transporter associated with antigen processing (TAP) consisting of the TAP1 and TAP2 subunits [57]. It is noteworthy that TAP can also transport significantly longer peptides into the ER, where the ER-resident aminopeptidases associated with antigen processing (ERAP)1 and ERAP2 trim these peptides to the appropriate length for MHC class I binding [58]. In the lumen of the ER, the assembly of the MHC class I heavy chain (HC) with the β_2_-microglobulin (β_2_-m) occurs, which is assisted by various chaperones serving as a quality control for proper folding and stabilization of the MHC class I dimer. These include calnexin, calreticulin, the oxido thiol reductase ERp57, and tapasin (tpn) yielding along with TAP the peptide loading complex (PLC) [59,60]. Furthermore, tpn proofreads peptides for stable binding in the MHC class I binding groove and facilitates their loading onto MHC class I molecules [61]. Upon a successful peptide loading, the trimeric MHC class I HC/β_2_-m/peptide complex is released from the PLC, and then transported via the trans-Golgi apparatus to the cell surface, where it can be screened by the TCR of CD8^+^ CTL [62]. Therefore, the proper expression of the major components of the complex MHC class I APM is obligatory for MHC class I surface expression and for effective T cell recognition of tumors [59,63,64].

CD4^+^ T cells are also important for mounting anti-tumor immune responses by recognizing Ags presented on MHC class II molecules of antigen-presenting cells (APCs), but also on tumor cells [63,65,66]. In contrast to MHC class I antigens expressed on all nucleated adult cells, the expression of the heterodimeric MHC class II surface molecules is more restricted and preferentially found on the cell surface of professional APCs [65]. However, accumulating evidence of tumor-specific MHC class II expression exists, which is associated with a favorable outcome in patients with cancer [67]. MHC class II surface expression can be induced in other cell types by various cytokines [68,69,70], in particular, IFN-γ. Exogenous Ags phagocytosed by APCs are directed to the endosomal compartments containing accessory chaperones and cathepsins that process and cleave Ag into small peptide fragments (Figure 2B) [71]. In the ER, MHC class II molecules assemble as dimeric proteins consisting of one α and one β chain and associate with the class II invariant chain (Ii, CD74), which prevents binding of ER-derived peptides [72]. Endosomal targeting signals facilitate trafficking of MHC class II molecules into a specialized endosomal compartment, where the Ii is degraded into the class II invariant chain peptide (CLIP) fragment by a series of key cleavage events, thereby protecting the MHC class II binding groove [73]. The loading of MHC class II molecules with exogenously derived peptides is assisted by the chaperone-like components HLA-DM and -DO, which facilitate the exchange in the CLIP fragment with these peptides [74]. In addition, HLA-DM edits the presented peptide repertoire catalyzing multiple peptide exchanges, which possibly favors the most stable complexes [75]. Then, the peptide-loaded MHC class II molecules are transported to the cell surface and presented to CD4^+^ T lymphocytes [76,77]. In professional APCs, exogenous antigens can also gain access to the MHC class I pathway through distinct cross-presentation mechanisms. Vice versa, the endosomal MHC class II loading pathway could also receive peptides derived from endogenous Ags through autophagy and other mechanisms [78,79]. 

### 3.2. Regulatory Elements of the Major Histocompatibility Complex Class I and II Antigen Processing Machinery Promoters and Their Interferon Inducibility

The expression of MHC class I and II APM components is tightly regulated in cells, has been well characterized, and exerts some similarities, but also unique properties [80]. It is mediated by promoters of the MHC class I and class II APM components; some of them contain TATA and CAAT boxes, whereas others completely lack these regulatory domains in their promoters (Figure 2C). In addition, TAP1 and LMP2 are transcribed from a shared bi-directional promoter of only 596 base pairs separating their ATG translation initiation codon [81]. The promoters of the major MHC class I APM components contain a combination of distinct transcription factor binding sites (TFBS), such as Sp1, CREB, the nuclear factor (NF)-ĸB, E2F, and p300 [82,83,84]. The constitutive MHC class I transcription is controlled by nucleotide-binding domain and leucine-rich repeat containing (NLR) family members, in particular, the NOD-like receptor caspase activation and recruitment domain (CARD) containing 5 (NLRC5), which is recently suggested to be one major regulator of the MHC class I transcription and upregulates the expression of some (β_2_-m, TAP1), but not all APM genes (TAP2) [85,86]. Furthermore, most MHC class I APM promoters have IFN response elements, which suggest their regulation by IRFs [86,87] (Figure 2C). Indeed, secretion of and/or treatment with type I, II, and III IFNs can upregulate MHC class I and/or class II surface expression by enhancing the transcription of MHC genes and APM components via IRF1 upon activation of STAT1 [85,88,89]. 

Regarding the MHC class II pathway, the promoters of the invariant chain, HLA-DM/-DO and the MHC class II HC contain an IFN-response element and similar, but also distinct TFBS in their promoters [84,90]. An exception is represented by the class II trans-activator protein (CIITA), the master regulator of the constitutive and inducible MHC class II expression, which is regulated by multiple promoters in different professional and non-professional APCs [30]. CIITA does not bind DNA, but interacts with the transcription factors RFX, NFY, and CREB and associated chromatin-modifying enzymes [91], thereby forming an enhanceosome governing the MHC class II transcription [92]. There are three tissue-specific CIITA promoters, namely CIITA-PI, -PII, and -PIII, which differ in their TFBS composition [93]. One promoter controls the constitutive CIITA expression in dendritic cells (DC), whereas another is specific for its constitutive expression in B cells [94]. In contrast, the CIITA-PIV regulates the induction of the CIITA expression in different cell types and contains several *cis* elements including a putative NF-ĸB site overlapping with an AP2 site, the IFN-γ activating sequence (GAS), the E box, and an IRF element [95,96,97]. STAT1 and IRF1 cooperate with the ubiquitously expressed trans-activating factor upstream stimulatory factor 1 to activate CIITA-PIV [98]. In addition to controlling CIITA, various MHC class II APM components are coordinately expressed due to common TFBS. Altogether, these data suggest that the activity of the different MHC class I and II APM component promoters can be induced by different members of the IFN family, but to a distinct extent. IFN-γ is the strongest inducer of the MHC pathways when compared to type I and type III IFNs. Interestingly, a combination of type I and type II cytokines exerts additive or even synergistic effects on MHC class I and II APM components [89].

In summary, the activation of the adaptive immune responses by IFNs, in particular, by IFN-γ, is mainly mediated by a transcriptional activation of MHC class I and class II antigens and diverse APM components [99,100]. These include MHC class I and II HC, β_2_-m, the invariant chain Ii, HLA-DM/-DO, CIITA, TAP, tpn, the LMPs, and ERAP1/2. Recently, the zinc finger transcriptional repressor Blimp-1 (PRDM1), originally identified as the silencer of IFN-β gene expression in virus-infected cells, has been shown to regulate the expression of components of the IFN signaling and MHC class I pathways [101] by directly targeting the transcriptional activator STAT1 [102].

### 3.3. Mechanisms of Cancer Cell Immune Evasion Mediated by Impaired Major Histocompatibility Class I and Antigen Processing Machinery Component Expression 

Decreased or absent expression of the MHC class I molecules has been observed with a high frequency in a broad number of solid human and hematopoietic tumors [103,104], such as, e.g., total or selective HLA haplotype allelic losses or downregulation of HLA class I molecules or loci have been identified supporting the role of HLA class I abnormalities leading to an escape from T cell-mediated immune surveillance [105,106,107]. The underlying mechanisms of total or partial loss of HLA class I antigens are diverse and include mutations of the β_2_-m gene [108] and loss of heterozygosity (LOH) of MHC genes (Table 2) [109]. Mutations in different APM components are not widespread and appear to be a rare event [110,111,112]. Since structural defects cannot be corrected by cytokine treatment, T cell-based therapies may not be effective due to the irreversible loss of HLA class I molecules. However, it is postulated that a deregulation rather than genetic abnormalities is the major cause for aberrant APM component expression (Table 2) [106,113,114]. This hypothesis is further supported by experiments that aimed at (i) characterizing the APM promoter activity in tumors, (ii) determining the epigenetic control, and (iii) posttranscriptional mechanisms regulating HLA expression, as well as (iv) treating tumors with modulators of the cytokine signal transduction and/or demethylating agents to determine whether deficiencies of APM component expression could be reverted by these treatments [115]. Indeed, the impaired MHC class I APM component expression of tumor cells could often be restored upon treatment with the different IFN family members, with IFN-γ being the most potent inducer [100]. The IFN-mediated upregulation of APM components often results in an enhanced MHC class I surface expression, which is required for the generation of an effective anti-tumor specific immune response [110,116]. Indeed, the IFN-induced upregulation of APM components improves anti-tumor specific CTL responses [117,118], and therefore represents a valuable strategy for the treatment of patients with transcriptional deregulated APM component expression. However, in some cases, tumors could remain insensitive to IFN treatment despite the lack of structural alterations in MHC class I APM components suggesting additional regulatory mechanisms, such as methylation, posttranscriptional or posttranslational regulation, as well as impaired IFN signal transduction [110].

## 4. Defects in the Interferon Pathways

### 4.1. Frequency of Defective IFN Inducibility of APM Components in Tumor Cells

Over the last decades, the molecular basis of IFNs, with the exception of type III, has been elucidated and their function in anti-tumoral activity, anti-viral, and pathogen defenses has been well characterized [119]. IFNs play a critical role in orchestrating innate and adaptive immune responses to tumors [120]. Importantly, impaired IFN signaling and/or unresponsiveness to IFN treatment is frequently found in different tumor types [121] including renal cell carcinoma (RCC), lung tumors, and melanoma, which was mainly determined for IFN-γ using in vitro tumor cell lines. Approximately 20–35% of melanoma and non-adenocarcinoma lung tumor cell lines had a reduced IFN-γ sensitivity, while ~10% of lung adenocarcinoma cell lines were resistant to IFN-γ treatment. These data were extended in a recent study, in which 57 melanoma cell lines were monitored for their capacity to upregulate MHC class I surface antigens in response to IFN-γ stimulation. In vitro resistance to IFN-γ treatment was found in <5% (2/57) of melanoma cell lines [122,123]. Interestingly, an impaired IFN-γ response despite a functional IFN-α induction and vice versa may exist, suggesting that the different IFN signal transduction cascades might not be coordinately regulated in tumor cells [5,124,125]. However, the number of tumor types and cell lines analyzed for IFN resistance is still limited, and further studies are required for better insights into the frequency, relevance, and molecular mechanisms of these deficiencies in tumors [126].

### 4.2. Mechanisms of Defective Interferon Inducibility of the Expression of Major Histocompatibility Complex Class I Antigen Processing Machinery Components in Tumors

To understand the underlying molecular mechanisms of impaired IFN inducibility, different experimental approaches were used over the years. In early studies, electro mobility shift assays demonstrated a differential binding of TFs to the ISRE of, e.g., MHC class I regulatory elements, which was responsible for the lack of MHC class I inducibility [127]. Furthermore, the IFN unresponsiveness was correlated with the expression profile of ISGs by performing cDNA micro-array analyses of IFN-sensitive vs. IFN-resistant cells using a customized microarray consisting of 850 ISGs [128]. These data demonstrate that the expression of ISGs associated with transcription precedes the expression of ISGs involved in signal transduction. Although no differences in the STAT1 induction were observed, subtle alterations in the expression profile might be responsible for the insensitivity to IFNs. Using this approach, the maintenance of the transcriptional activation upon IFN treatment appeared to enhance IFN sensitivity.

The importance and involvement of IFN signal transduction pathways in the transcriptional regulation of MHC class I and II antigens has been established, but there is only limited information about the underlying molecular mechanisms of the lack of IFN inducibility of APM component expression [124,126,129,130]. This could occur at different steps in the IFN signal transduction pathways and might involve both sequence abnormalities and/or different regulatory processes, such as transcriptional, post-transcriptional, and epigenetic control (Figure 1; Table 3).

The physiological relevance of the STAT/JAK pathway in the regulation of MHC class I and II antigens has first been established in mice and cell lines with a targeted disruption of these genes [131,132,133,134]. The impaired JAK1 and JAK2 activity was associated with a loss in the capability of IFN-γ to induce growth arrest and apoptosis and enhance immunogenicity mediated by the lack of APM induction in tumor cells [135]. Based on the current knowledge regarding the transcriptional regulation of the dual TAP1 and LMP2 promoter, the loss of TAP1 and LMP2 expression may be attributable to deficiencies of STAT1 and IRF1, which might be also linked to an altered growth inhibition [121,122,128,135,136]. The IFN-α resistance of RCC cell lines has been associated with a defective induction of STAT1 that could be restored by the addition of supernatants from PMA-stimulated peripheral mononuclear cells [136], in particular, secreting IFN-γ, although other cytokines might be also involved in this process.

The loss of the IFN-γ-mediated upregulation of MHC class I APM components in some RCC cell lines appears to be due to the lack of IRF1 and STAT1 binding activities upon IFN-γ stimulation. The STAT1, JAK1, and JAK2 proteins were expressed in RCC cell lines, but not phosphorylated in the presence of IFN-γ [137]. Furthermore, an association of impaired STAT1 phosphorylation with the loss of IFN-mediated HLA class I induction was also found in melanoma cell lines [122]. The absence of STAT1 phosphorylation was at least partially due to the constitutive expression of the suppressor of cytokine signaling (SOCS)1 protein and vice versa [138]. SOCS1 modulates the IFN-γ-mediated signaling by binding to the autophosphorylation site of JAK2 and by targeting bound JAK2 to the proteasome for degradation [139]. SOCS1 overexpression has also clinical relevance and correlates with melanoma progression by conferring a growth advantage [140,141]. In another study, IFN-γ resistance was linked to constitutive SOCS3 expression [142]. The expression of IFN-γ-responsive genes is also reduced in the choriocarcinoma cells JEG3 and JAR in comparison to the epithelial cell line Hela [143], which is mediated by a compromised tyrosine phosphorylation of JAK2 and STAT1 at Tyr-701 and a reduced expression of IRF1. In addition, inhibition of the tyrosine phosphatases results in increased JAK1 and STAT1 phosphorylation and IFN-γ-induced gene expression in these cells [143]. An impaired expression of IRF1 and deficient phosphorylation of STAT1 was also observed in primary trophoblast cell lines, suggesting that these defects are of clinical relevance.

In addition to the post-translational regulation of components of the IFN signal cascades, the absence of the IFN-γ-mediated MHC class I expression can be controlled by epigenetic alterations of the IFN pathway. Indeed, promoter methylation of target genes affects the binding of IRF1, leading to an abrogation of the IRF1 transactivation [122]. Treatment with the demethylating agent 2′5′-deoxyazacytidine (DAC) restored the IRF1 expression and consecutively led to the reconstitution of the IFN-γ-mediated MHC class I inducibility [129]. This is in line with other studies postulating that the IFN unresponsiveness is attributed to the low expression of STAT1 due to its promoter methylation rather than to an absent phosphorylation [144,145].

Finally, there is evidence that the genetic instability of tumor cells can modulate/inhibit the expression of the IFNGR, which in some cases might be associated with cancer prognosis. For instance, the loss of IFNGR independently predicts the poor prognosis of ovarian cancer patients and might be responsible for the limited success in the outcome of IFN-γ treatment of ovarian cancer [146]. Transduction of IFN-λ into tumor cells induced MHC class I expression and inhibited tumor growth. This was associated with an increased lymphocytic infiltration, suggesting that the delivery of IFN-λ might be a possible treatment strategy [147].

### 4.3. Involvement of the Interferon Pathways in Tumor Surveillance In Vivo

Multiple activities of IFNs on tumor cells might coordinate the anti-tumor immune responses by affecting the activity of immune cells, in order that the early recognition and/or elimination of cancer cells by innate immune cells result in an immune attack by the adaptive immune system [148]. In addition, IFN-γ treatment influences the tumor cell immunogenicity and mediates immune responses, which are directed against tumor cells through distinct mechanisms. For example, IFN-γ can downregulate the expression of NKG2D ligands, and at the same time, increases the expression of MHC class I molecules [149]. In vitro treatment with IFN-γ decreased the elimination by NK cells independent from the expression of HLA class I molecules, whereas an upregulated MHC class I expression enhanced the sensitivity to CTL-mediated lysis. In addition, these in vitro results of abnormalities in the IFN signaling can occur in vivo. LMP2^−/−^ mice exhibited an impaired proteasome function and 36% of female LMP2^−/−^ mice developed uterine leiomyosarcoma by 12 months of age. Therefore, the development of spontaneous human uterine leiomyosarcomas might be probably due to defects in early steps of the IFN signal cascade. Indeed, the defective TAP1 and LMP2 expression in these tumors is associated with a G871E mutation in the ATP-binding region of the JAK1 kinase domain, thereby affecting JAK1 kinase activity, but neither JAK1 expression nor its degradation [130] allow the tumor cells to evade anti-tumor specific immunity. In different cancers, immunosuppression associated with STAT3 activation and STAT3-mediated inhibition of DC function has been reported [26]. The biological function of STAT1 and STAT3 differs in terms of cell growth and induction of an anti-tumor immune response. Whereas STAT1 abrogates growth and mediates anti-tumor effects, STAT3 promotes cell proliferation and tumorigenicity as it has been shown in melanoma and head neck squamous cell carcinoma. In both tumor entities, STAT3 expression is associated with tumor progression and mediates immune suppression. In addition, unphosphorylated or phosphorylated STAT1 and STAT3 are coordinately upregulated by both IFN-α and IFN-γ and may represent a marker for the dynamic mechanism of melanoma progression and host response. Using untreated and methylcholanthrene treated IFNγR^−/−^ mice, a significantly higher tumor development was observed in the IFNγR expressing control mice. The crossing of IFNγR1 and STAT1^−/−^ mice with p53^−/−^ mice resulted in a spontaneous and more rapid tumor development, in particular, teratomas, hemangiomas, and chondrocytomas. Interestingly, the IFN-γ-sensitive tumor cells transfected with the dominant negative IFNγR mutant grew faster compared to the untransfected tumors and were not rejected upon treatment with lipopolysaccharide, which effectively eliminated the control of tumors [150]. Furthermore, downregulation of the IFNγR is associated with the loss of Fas function and is linked to tumor progression [151]. Therefore, the IFN-γ responsiveness is an important mechanism in the control of tumor growth. An increased responsiveness to metastases promoting agents might be induced by many mediators of the tumor microenvironment (TME) including type I and type II IFNs. Both cytokines cooperate with TNF-α, which involves a positive interplay between JAK1 and PKC signal transduction [152]. Furthermore, IFN-α/β elicit differential inflammatory responses depending on the cellular localization. Therefore, the IFN-mediated effects are driven by tissue identity and can be shaped by the immunological context, in which the IFNs are produced. These data suggest that multiple signals can be generated by the host inflammatory cells, which cooperate with the invasive properties of tumor cells. Therefore, strategies targeting this cross talk between tumor and host cells in the microenvironment are needed to prevent tumor growth.

In this context, it is noteworthy that IFN-γ not only promotes the expression of immune stimulating, but also immune inhibitory molecules, such as Fas/Fas-L, PD-L1, PD-L2, and IDO1, which are known to limit anti-tumor responses [151,153].

### 4.4. Effect of Interferon Signaling on the Expression of Major Histocompatibility Complex Class II Components in Tumors

A link between the oncogenic pathways, HLA class II antigens and IFN signaling has been suggested. For example, the chimeric RET/PTC (rearranged in transformation/papillary thyroid carcinoma) oncoproteins were constitutively expressed in papillary thyroid cancer and were able to phosphorylate the Tyr-107 of STAT1, which is accompanied by IRF1 expression [154]. This is also associated with an enhanced transcription of CIITA and consequently of MHC class II expression in the papillary thyroid carcinoma cells, thereby explaining the increased immune cell infiltration of RET/PTC-positive cancers. Furthermore, a synergistic activity of TNF-α and IFN-γ on CIITA was found in thyroid carcinoma [155]. The CIITA-controlled MHC class II expression in human cells could be upregulated by histone deacetylases, such as trichostatin A (TSA) [156,157]. However, CIITA was refractory to IFN induction in many human tumors. In melanoma, colorectal, and gastric carcinoma, CIITA is silenced by epigenetic mechanisms resulting in the lack of IFN-γ-induced MHC class II expression [125,158]. 

### 4.5. Inborn Errors of Type I Interferon Immunity and Their Phenocopies among Humans

Insights into the critical roles and also redundancy of type I IFN immunity in human health and disease have come from numerous in-depth studies of patients with germline loss-of-function mutations in various genes involved in type I IFN signaling [159]. Human IRF7 deficiency, impairing the inducible transcription factor responsible for amplifying type I IFN responses, was first discovered in an otherwise healthy child with life-threatening influenza [160]. Similarly, IRF9 deficiency, which impairs the DNA-binding component of the interferon-stimulated gene factor 3 (ISGF-3) complex, was also first identified in a child with severe influenza pneumonitis [161]. Surprisingly, several previously healthy adults with critical COVID-19 were found to have autosomal recessive (AR) complete IRF7 or IFNAR1 deficiency, autosomal dominant (AD) TLR3, UNC93B1, TICAM1, TBK1, IRF3, IRF7, IFNAR1 or IFNAR2 deficiencies, or X-linked TLR7 deficiencies, establishing type I IFN immunity as a critical factor to control infection with SARS-CoV-2, while being largely redundant in the context of various other infections [162,163]. Notably, X-linked recessive TLR7 deficiency alone accounts for approximately 1% of critical cases of COVID-19 in male patients under the age of 60 years [164]. In contrast, individuals with a complete AR IFNAR1 and IFNAR2 deficiency are rare (reviewed in [159]). Complete loss-of-function (LOF) of one of the subunits of the heterodimeric IFNAR complex has also been associated with disseminated infection from live attenuated measles, mumps, and rubella (MMR) or yellow fever vaccines (YFV) in infancy, as well as in one case with fatal Herpes simplex virus encephalitis (HSE) [165,166]. Gene defects that disrupt TLR3-dependent IFN-α/β immunity are also well-established risk factors for HSE in children (reviewed in [167]). Another remarkable and unexpected discovery during the recent SARS-CoV-2 pandemic was the high prevalence of type I IFN neutralizing auto-Abs in convalescent COVID-19 patients, which was strongly associated with critical illness, affected more men than women, and increased with age accounting for approximately 10 to 20% of severe cases [163,168]. More recently, type I IFN-neutralizing auto-Abs were also found among approximately 5% of a cohort of 279 pediatric and adult patients with critical influenza pneumonia suggesting a similar pathological role [169]. Of note, type I IFN neutralizing auto-Abs are also found in the general population with a similar bias toward men and increasing age, but with a significantly lower prevalence than among convalescent COVID-19 patients [163,168]. Whether any of these germline-genetic or immunological factors that impair type I IFN immunity are also associated with increased cancer risk remains to be investigated.

### 4.6. Inborn Errors of Type II Interferon Immunity

Complete IFN-γ deficiency, IFNGR1 or IFNGR2 deficiency, and a number of other inborn errors of type II IFN immunity underlie Mendelian susceptibility to mycobacterial disease (MSMD) in humans, which is characterized by selective susceptibility to weakly virulent mycobacteria, such as the Bacille Calmette-Guerin (BCG) vaccine substrain and environmental mycobacteria, as well as to more virulent species of the *Mycobacterium tuberculosis* (Mtb) complex [170]. Indeed, with the exception of ZNFX1 deficiency (ZNFX1 is a conserved and broadly expressed helicase that is recruited to, or induces stress granules), for which the mechanism of disease remains to be determined [171], all of the currently known monogenic etiologies of MSMD (which encompass mutations in as many as 18 genes and an even greater number of disorders due to the high degree of allelic heterogeneity) impair the production of, or cellular responses to IFN-γ [170,172]. The same is true for rare inborn errors underlying tuberculosis in humans, including complete IL-12Rβ1 deficiency, certain TYK2 deficiencies, interleukin-2 inducible T cell kinase (ITK) deficiency, and PD1 deficiency. These conditions lead to impaired IFN-γ production by multiple lymphocyte subsets, thereby resulting in an insufficient protection against Mtb, but sufficient immunity to BCG and environmental mycobacteria [173,174,175,176,177]. Of note, the common TYK2 P1104A missense variant underlies tuberculosis in approximately 1% of patients with European ancestry and is associated with an impaired IL-23-dependent IFN-γ immunity [178,179]. In conclusion, while mice lacking IFN-γ were shown to be susceptible to infection by both viruses and bacterial pathogens capable of surviving macrophage-mediated phagocytosis [180,181], the study of MSMD patients has shown that human IFN-γ is primarily a macrophage-activating factor and appears less important for anti-viral immunity. Notably, while both type I and II IFNs induce the transcription factor IRF1, in vitro studies of cells obtained from unrelated children with inherited complete IRF1 deficiency and multiple, life-threatening diseases in early life caused by weakly virulent mycobacteria and related intra-macrophagic pathogens but no history of severe viral disease demonstrated that IRF1 is essential for IFN-γ-dependent macrophage immunity to mycobacteria, but largely redundant for type I IFN-dependent anti-viral immunity [182]. A recent study of a patient with inherited PD-1 deficiency and TB, who died of pulmonary autoimmunity, has also highlighted the indispensable role of human PD1 in governing both anti-mycobacterial immunity and self-tolerance and provided important mechanistic insights relevant for the treatment of cancer patients who receive immunotherapy with PD-1-directed checkpoint inhibitors, thereby exhibiting a high vulnerability to tuberculosis for previously unknown reasons. The study showed that the PD-1 deficiency in the patient undermined the IFN-γ production by T lymphocytes and NK cells and caused lymphoproliferative autoimmunity through over-activating STAT3 in ROR-γT expressing DN αβ T cells [175].

Neutralizing anti-IFN-γ auto-Abs are also associated with disease, specifically with an adult onset and ethnicity-biased immunodeficiency, which is characterized by a susceptibility to infectious diseases caused by obligate intracellular bacterial and fungal species that have the capacity to survive, and some to even replicate within the phagocytic compartment of macrophages. These include disseminated environmental mycobacteria disease, disseminated tuberculosis, salmonellosis, caused by *Salmonella enteritidis D* or *Salmonella typhi*, cryptococcosis principally caused by *Cryptococcus neorformans*, histoplasmosis caused by *Histoplasma capsulatum,* and talaromycosis caused by *Talaromyces* [*Penicillium*] *marneffei* [183]. However, the latter is significantly less frequent than neutralizing anti-type I IFN Abs and associated diseases, such as severe COVID-19 or critical influenza pneumonia [169,183].

### 4.7. Human Autosomal Recessive Complete STAT1 and STAT2 Deficiencies

Since STAT1 participates in transcriptional regulation upon activation of both IFNAR and IFNGR, cells from patients with AR complete STAT1 deficiency totally lack response to type I, II, and III IFNs, as well as IL-27. Clinically, these patients suffered from multiple episodes of severe viral infections as well as from syndromic MSMD. In contrast, patients with complete AR STAT2 deficiency typically present with disseminated infection from live attenuated vaccines and recurrent viral infections, with incomplete penetrance, since STAT2 does not participate in signaling upon engagement of the IFNGR (reviewed in [159]).

### 4.8. Major Histocompatibility Complex Deficiencies in the Context of Infection and Tumors

Null alleles for the classical HLA genes are increasingly identified [184], mainly in population genetic studies [185] and are typically not causal for severe disease by themselves. This is due to the high degree of immunological redundancy of the classical class I glycoproteins, namely HLA-A, -B, and -C, and of the classical class II glycoproteins, HLA-DR, -DP, and -DQ [186,187,188]. Each of these glycoproteins participate in antigen presentation through the engagement of the αβ antigen receptors on cytotoxic (CD8^+^) T cells and helper (CD4^+^) T cells, respectively. In contrast, HLA class I and II deficiencies due to LOF mutations in genes encoding regulatory or accessory factors that control the assembly of MHC molecules typically leads to CD8^+^ T cell and CD4^+^ T cell lymphopenia, respectively, and combined immunodeficiencies with a variety of clinical manifestations [189]. Furthermore, the downregulation or loss of MHC-I molecules due to somatic mutations are common immune escape mechanisms in many solid and hematopoietic cancer types [190]. 

## 5. Clinical Relevance of Interferons

### 5.1. Clinical Relevance of Aberrant Interferon Signaling in Tumors and Upon Viral Infection

IFNs have been used in various clinical settings, since they are potent negative regulators of cell growth by modulating the cell cycle or by inducing pro-apoptotic genes [191,192,193,194]. In addition, the importance of IFNs in maintaining immune homeostasis resulted in their therapeutic applications for shaping innate as well as adaptive immune responses. Type I IFNs have been demonstrated to inhibit tumor growth directly or indirectly by acting on immune and tumor cells as recently reviewed [195]. IFN-α has been extensively used for the treatment of various malignancies during the last two decades, demonstrating an improved clinical outcome of hematological malignancies (chronic myeloid leukemia (CML), cutaneous T cell lymphoma, hairy-cell leukemia (HCL), multiple myeloma, solid tumors including malignant melanoma, RCC, AIDS-related Kaposi’s sarcoma, and viral syndromes (hepatitis C, hepatitis B, severe acute respiratory syndrome, and COVID-19) [196,197]. IFN-γ has shown positive results in the treatment of chronic granulomatous disease, multiple sclerosis, and severe malignant osteoporosis [198], whereas it was of limited success, in particular, in oncology [199]. Recent reports even demonstrated a pro-tumorigenic effect of IFN-γ [200], suggesting a dual function of this cytokine. Concerning cancers, resistances to IFNs have been described, which limit their anti-cancer activity. These were correlated with defects in the IFN signaling resulting in the impaired expression of IFN-responsive genes, which might not only have important implications for immunotherapies, but also for transplantations, pregnancy, and for the development of tumors. Despite proven clinical efficacy in the treatment of malignancies, viral infections and multiple sclerosis, a substantial number of patients lack a clinical response to IFN therapy. For example, IFN-α2b is a clinically active therapeutic agent for malignant melanoma and RCC, only 15 to 20% patients with metastatic melanoma respond to IFN therapy [201,202]. Other reviews report even lower response rates of only 6% in treated melanoma patients [203,204]. In RCC, the best results of IFN treatment determined by the response rate and duration of the effect were obtained in patients with a good functional state after nephrectomy without chemotherapy and with preferentially lung metastasis [205]. The survival rate of these patients increased from 49 to 115 weeks upon IFN-α administration. Since none of these factors has been proven to be associated in an unambiguous way with the cytokine response and the patients’ survival, there is an urgent need to identify biomarkers for the selection of patients who might benefit from this therapy. In contrast to type I IFNs, IFN-γ and IFN-λ have not been approved for cancer treatment by the FDA. IFN-γ exhibits different direct effects on tumor cells during the anti-tumor immune response, supporting its relevance in the cancer immunoediting process [148]. Therefore, IFN-γ plays a central role in promoting natural immune responses directed against developing tumors [199,206,207]. However, its application in immunotherapeutic protocols has been very limited. In clinical trials, an IFN-γ treatment improved the survival in patients with ovarian cancer of stage Ic-IIIc [208], upon intra-vesical administration to patients with transitional-cell bladder carcinoma [209] or used for isolated-limb perfusion of individuals with non-melanoma cancers of the extremities [210]. In contrast, no effects were detected upon IFN-γ treatment of patients with metastatic RCC [211], advanced colon cancer [212], or small-cell lung cancer [213]. The limited therapeutic success of IFN-γ might reflect the inability to target IFNs to the right place with an efficient concentration [148]. Despite the proven pivotal role of endogenously produced IFN-γ in anti-tumor immunity in experimental in vivo models, the low response rate to this cytokine in cancer immunotherapy trials might be explained by the resistance of tumor cells to IFN-γ [121,122,214]. Targets of the immunologic unresponsiveness represent genes encoding components of the MHC class I APM or IFNGR signaling pathway [215,216]. The physiological relevance of HLA class I surface expression during the tumor rejection process in patients receiving different protocols of immunotherapy was assessed in two studies [217,218]. In the first trial, a significant difference in the immunotherapeutic response of patients exhibiting metastases with low levels of MHC class I surface antigens and those with high levels of MHC class I expression was detected. The second study demonstrated an impact of the cytokine unresponsiveness by determination of HLA class I antigen expression levels on metastatic melanoma lesions during the course of the disease in one patient undergoing IFN-α2b and autologous vaccination plus BCG (MVAX). BCG triggers the IL-12/IFN-γ axis and induces upregulation of genes associated with antigen presentation [219,220]. The level of the MHC class I antigen expression was dependent on the IFN response, since none of the progressor metastases increased the expression of HLA class I antigens after vaccination. However, a significant increase in the HLA class I surface expression was detected in the regressor metastases suggesting that HLA class I surface antigens on tumor cells significantly contributed to the therapeutic effect of BCG. This is in line with the observation that the downregulation of HLA class I surface antigens in cancer cells is a significant risk factor for recurrence in patients with intra-vesical BCG immunotherapy for bladder cancer [221]. Furthermore, defects in tumor-intrinsic IFN signaling results in failure of ICPi treatment due to upregulation of the expression of multiple genes involved in T cell exhaustion including, but not limited to PD-L1 [222], while IFN signaling could also potentiate the efficacy to ICPi [123,223].

Based on these results, a better understanding of the molecular mechanisms, by which tumors modulate the cytokine signaling, may be essential for the development of immunotherapeutic strategies with the aim to enhance MHC class I surface antigen expression in tumor cells. The balance of STAT phosphorylation versus SOCS expression might be crucial in the activation of immunologic responses through APM and MHC class I transactivation [224]. For instance, the effects of high dose IFNs are associated with immunologic processes, such as an upregulation of TAP1, TAP2, tpn, and LMP2. The STAT1 and STAT2 pathways in melanoma cells are sensitized to IFN-α by pretreatment of the cells with IFN-γ. Therefore, the biological response to IFN-α might be mediated by a direct effect on melanoma cells, suggesting a potential role for IFN-γ in the treatment of this disease [225]. In addition, IFN-α treatment of patients with cutaneous melanoma significantly modulates the balance of STAT1/STAT3 in tumor cells and host lymphocytes. This results in an upregulation of TAP2 and an increased immune response [224].

### 5.2. Other Clinical Applications of Interferons

The current FDA-approved IFN therapy of tumors is the application of IFN-α for the treatment of hematological malignancies, such as HCL, chronic myeloid leukemia (CML), as well as follicular lymphoma. In addition, some solid tumors, such as melanoma, RCC, and hepatocellular carcinoma, have been treated with IFNs. For HCL, IFN-α was successfully employed, while for CML and follicular lymphoma, IFN has been shown to control disease, but exhibit substantial toxicities. In melanoma, IFN-α treatment improved the outcome of patients with stage 2B and 3 melanoma leading to a prolonged relapse free-survival and overall survival of these patients [226]. Based on experimental murine models, there is accumulating evidence of potential links between type I IFN expression signatures, T cell infiltration, and clinical outcome suggesting their use in combination with immunotherapies [195,227,228,229]. This includes, for example, the treatment of murine models of colon carcinoma with, e.g., STING agonists, in order to modulate the TME [195,230]. Furthermore, first clinical trials combining ICPi monotherapy with IFN-α treatment have been carried out in melanoma patients with promising results [231]. In addition, treatment with IFN-γ generated disappointing results in the clinics with only some tumor types, such as ovarian and bladder cancer responding to treatment [208,209], whereas BC and RCC did not [211,232]. Different studies are being investigated to enhance clinical efficacy, but inhibiting side effects, such as the development of recombinant forms with enhanced IFN-γ half-life or partial agonist activities, a more tissue-specific homing, as well as viral and non-viral genetic approaches to induce IFN-γ expression in situ [233,234,235]. In addition to the treatment with type I and type II IFNs, first preclinical data exist on the application of type III IFNs. Indeed, the in vivo tumor growth of different murine tumor cells, such as B16, CT26 colorectal cancer, and MCA2005 fibrosarcoma cells, could be reduced upon overexpression or local induction of IFN-λ [236,237,238]. Mechanistically, this was due to both direct effects of IFN-λ on tumor cells, but also to the modulation of effector cells from innate as well as adaptive immunity, such as NK cells and CD8^+^ T cells [158,239]. 

In the context of infectious diseases, the use of type I IFNs as a treatment for selected COVID-19 patients and risk groups have been the focus of several clinical trials [240], although only a few of them were rigorously conducted and randomized double-blind placebo-controlled trials, making the interpretation difficult due to a variety of factors, most notably the relatively late administration of IFNs in the course of infection [241]. Similarly, although IFN-λ has been considered a promising therapeutic option for the treatment of TB patients, particularly of multiple drug resistant TB patients, further studies of its effectiveness in controlled clinical trials are required [242]. Since auto-Abs neutralizing type I IFNs apparently underlie a considerable proportion of deaths from COVID-19 worldwide, detection of these auto-Abs before, or early in the course of, infection could be used to identify individuals at risk and prioritize them for vaccination campaigns [163]. 

### 5.3. Immunotherapy Resistance—Association with Defects in the IFN Pathway

Despite the good results obtained with tumor immunotherapy strategies, still a high percentage of patients lack response to this treatment. In search for biomarkers of response versus resistance to therapy, in particular, to ICPi, an important role has been described for IFN signaling and the induction of IFN-γ gene signatures [243]. This is in accordance with a recent report demonstrating that IFN-γ signaling is driving the clinical responses to ICPi [123]. Furthermore, a panel of stimulatory genes (STAT1, MX1, SG15, OAS1, IFIT1, IFIT3, IFI44, and USP18) was associated with response to therapy. Moreover, adaptive immune resistance mediated by defects in IFN signaling caused a downregulation of IFN-γ, STAT1, JAK1, JAK2, chemokine receptors, and ligands (CXCL9, CXCL10, CXCL11, CCR5), IDO1, PRF1, and HLA class I, which have been identified as members of an IFN signature. In addition, loss of function mutations or downregulation have been described in major IFN and MHC class I APM components, such as HLA class I, HC, β_2_-m, TAPs, JAK2, IRFs, JAK1, and STATs, resulting in the lack of response to IFN and ICPi therapy [244]. Furthermore, tumor-intrinsic IFN-γ signaling has shown to shape tumor-infiltrating lymphocytes and influence ICPi resistance of melanoma with defective IFN-γ signaling [245]. Similarly, to identify strategies for overcoming defective IFN signaling, cells lacking JAK1 and JAK2 expression were generated using CRISPR/Cas9 editing and their response to therapy was analyzed. Treatment with the TLR9 agonist has been shown to initiate a potent type I IFN response, thereby overcoming anti-PD1 resistance in JAK1-/JAK2-deficient mouse models. Interestingly, an induction of type I IFN could induce a strong and productive inflammation, while chronic type I IFN production is responsible for immune suppression and therapy resistance [246]. Age and sex might also play a significant role in the induction of (innate) immune responses and susceptibility to response to immunotherapies.

## 6. Conclusions

An increased knowledge of the factors responsible for the resistance to IFNs might lead to an improved use of these cytokines in malignant diseases. The application of the molecular analysis of tumor tissues have provided insights into tumor phenotypes and prognostic variables, leading to an optimization of specific treatment programs and patients’ selection. The identification of tumor lesions with the capacity to upregulate MHC surface expression will determine the ability to present new antigenic peptides to T lymphocytes, favoring regression of primary or metastatic tumor lesions. In contrast, tumors with irreversible genetic lesions associated with loss of MHC surface expression will not be able to efficiently present new antigenic peptides to T cells, thereby subsequently favoring tumor and/or metastases progression. We propose that suppression of IFN signaling in tumors contributes to tolerance by inhibiting the expression of genes encoding subunits of MHC class I/II antigens and/or components of their APM, which could be detrimental to successful anti-tumor responses. Despite the fact that studies focusing on the multifaceted mechanisms regulating the IFN response have impacted the design of immunotherapies, further investigations on the development of tailored IFN-based therapies are required in various diseases.

## Figures and Tables

**Table 1:** Features of the three interferon families and their characteristics.

	Type I	Type II	Type III
	IFN-α	IFN-γ	IFN-λ
chromosomal localization	9p21	12q14	19q/3
numbers	13 α, β, ε, κ, ω in humans	1	4
receptor	IFN-αRI IFN-αRII ubiquitously expressed	IFN-γRI IFN-γRII ubiquitously expressed	IFN-λR1 IFN-10Rβ preferentially expressed on epithelial cells
Signal Transduction pathways	JAK1, TYK2, STAT1–5, PI3K, AKT, MAPK, NF-ĸB, p53	JAK1, 2, STAT1, 3, 5, PI3K, AKT, MAPK, NF-ĸB	JAK1, STAT1–5, TyK2
main function	anti-viral anti-proliferative	anti-bacterial anti-proliferative	anti-tumoral anti-proliferative

This table summarizes the major characteristics of human type I, II, III IFN family members, their gene localization, the components of the receptor complex, and the signal transduction pathways involved.

**Table 2:** Mechanisms of impaired major histocompatibility complex class I expression.

Categories of Lesions	Reversibility by IFN	Mechanisms
(a) irreversible or “hard” genetic lesions:	no	total, locus- or allele-specific loss of the MHC class I HC LOH of the MHC class I HC mutations, deletions, recombination of β_2_-m structural alterations in LMP2, TAP1, TAP2, and tpn genetic abnormalities in JAK, STAT, and IRFs
(b) irreversible or “soft” lesions:	no	direct methylation of MHC class I HC, β_2_-m, tpn, NLRC5, and IRFs posttranscriptional downregulation of MHC class I APM components (miRNAs, RBPs) posttranslational modifications of APM components, e.g., TAP (phosphorylation) defects in the MHC class I export impaired IFN signal pathway (JAK, STAT)
(c) reversible or “soft” lesions:	yes	transcriptional downregulation of MHC class I HC gene expression selective MHC locus downregulation transcriptional downregulation of APM and IFN signaling components

**Table 3:** Defects in interferon signaling components associated with tumors and pathogen infections and interferon resistances.

	Molecule	Mechanisms
receptor	IFN-R expression	loss
signal transduction	JAK/STAT/TYK2	mutation, deletion
	JAK/STAT	lack of activity
	STAT1, STAT3, JAK1, and JAK2, SOCS1/3 altered expression	loss or aberrant phosphorylation
transcription factors	IRFs	reduced expression
	IRF1	impaired binding
	IRF1	methylation

## Data Availability

Not applicable.

## Abbreviations

Ab, antibody; AD, autosomal dominant; Ag, antigen; APC, antigen presenting cell; APM, antigen processing machinery; AR, autosomal recessive; BCG, Bacillus Calmette-Guérin; β_2_-m, β_2_-microglobulin; CIITA, class II transactivator; CIITA-PIV, class II transactivator protein promoter IV; CLIP, class II invariant chain peptide; CML, chronic myeloid leukemia; CTL, cytotoxic T lymphocytes; DAC, 2′5′-deloxyazacytidine; DC, dendritic cell; ER, endoplasmic reticulum; ERAP, ER-resident aminopeptidases associated with antigen processing; GAS, IFN-γ-activating sequence; HC, heavy chain; HCL, hairy cell leukemia; HSE, Herpes simplex virus encephalitis; ICPi, immune checkpoint inhibitor; IFN, interferon; IFNR, interferon receptor; Ii, invariant chain; IL, interleukin; IRF, interferon regulatory factor; ISG, interferon stimulated genes; ISGF, IFN-stimulated gene factor; ISRE, IFN-stimulated response elements; ITK2, interleukin-2 inducible T cell kinase; JAK, janus kinase; ITK, interleukin-2 inducible T cell kinase; LMP, low molecular weight protein; LOF, loss of function; LOH, loss of heterozygosity; MHC, major histocompatibility complex; MMR, measles, mumps, and rubella; MSMD, mycobacterial disease; Mtb, mycobacterium tuberculosis; NF-ĸB, nuclear factor ĸB; NLR, nucleotide-binding domain and leucine-rich repeat containing; NLRC5, NOD-like receptor caspase activation and recruitment domain (CARD) containing 5; PLC, peptide loading complex; RCC, renal cell carcinoma; RET/PTC, rearranged in transformation/papillary thyroid carcinoma; SARS-CoV-2, severe acute respiratory syndrome coronavirus type 2; SOCS, suppressor of cytokine signaling; STAT, signal transducer and activator of transcription; TAP, transporter associated with antigen processing; TB, tuberculosis; TCR, T cell receptor; TFBS, transcription factor binding sites; TLR, toll-like receptor; tpn, tapasin; TSA, trichostatin A; YFV, yellow fever vaccine.
